# When First‐Line Therapies Fail: Surgical Site Infections and the Declining Antimicrobial Utility in a Resource‐Limited Nation

**DOI:** 10.1002/mbo3.70312

**Published:** 2026-05-11

**Authors:** Innocent E. Nwachukwu, Cary W. Rivera, Vivian M. Reyes, Diomar Salazar, Lydia Harris‐Thurton, Danladi C. Husaini

**Affiliations:** ^1^ Department of Allied Health (Medical Laboratory Science), Faculty of Health Sciences University of Belize Belmopan Central Campus, Central America Belize; ^2^ Antimicrobial Resistance (AMR) Study Group, Allied Health Department, Faculty of Health Sciences University of Belize Belmopan C.A Belize; ^3^ Department of Allied Health (Pharmacy & Public Health), Faculty of Health Sciences University of Belize Belmopan Central Campus, Central America Belize

**Keywords:** antimicrobial resistance, antimicrobial stewardship, Belize, ESBL‐producing Enterobacteriaceae, MRSA, surgical site infections, treatment gaps

## Abstract

Antimicrobial resistance (AMR) poses a critical threat to surgical care in resource‐limited settings. This study describes the epidemiology of AMR in surgical site and wound infections in Belize from 2018 to 2024. Methods: A retrospective analysis of national laboratory data focused on *Escherichia coli*, *Klebsiella pneumoniae*, and *Staphylococcus aureus* from surgical and wound specimens. Resistance phenotypes (ESBL, MRSA), susceptibility profiles, and temporal‐spatial trends were analyzed. Results: Among 850 isolates, ESBL production among Enterobacteriaceae more than doubled from 25% to > 45% over 7 years, while MRSA remained persistently elevated (55%–65%). Critical treatment gaps emerged: over one‐third of ESBL isolates showed resistance to both oral (ciprofloxacin) and intravenous (gentamicin) options. ESBL infections clustered in central Belize and following cesarean sections; MRSA predominated in diabetic amputations. Antibiotic consumption preceded resistance by approximately 6 months. Sentinel carbapenem‐resistant Enterobacteriaceae and vancomycin‐resistant Enterococcus were detected. Conclusion: AMR is prevalent in Belize, with rapidly expanding ESBL rates and significant therapeutic limitations. Urgent interventions are needed, including updated treatment guidelines, targeted antimicrobial stewardship, and enhanced infection control in high‐burden areas.

## Graphical abstract

In Belize, a 7‐year nationwide study reveals a sharp rise in antimicrobial resistance in surgical infections. ESBL‐producing bacteria more than doubled to > 45%, with critical treatment gaps and geographic hotspots identified. Sentinel pan‐drug‐resistant cases and a correlation between antibiotic use and resistance underscore the urgent need for updated guidelines and targeted stewardship.

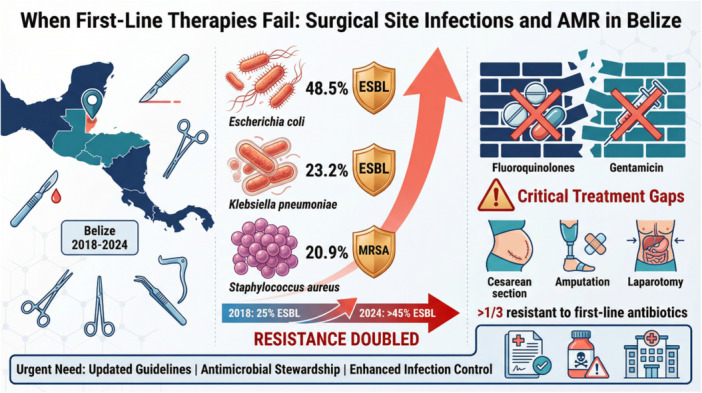

## 1 Introduction

Antimicrobial resistance (AMR) has evolved into one of the most formidable global public health and development threats of the modern era, with an estimated 1.27 million deaths directly attributable to resistant infections annually (World Health Organization WHO 2023). The relentless spread of multidrug‐resistant organisms (MDROs) fundamentally undermines the efficacy of modern medicine, jeopardizing treatments for common infections, the safety of surgical procedures, and the management of chronic diseases. This silent pandemic carries profound consequences, manifesting as longer hospital stays, exorbitant healthcare costs, and increased morbidity and mortality. In Central America and the Caribbean, the challenge is particularly acute, with common pathogens such as *Escherichia coli*, *Staphylococcus aureus*, and *Klebsiella pneumoniae* exhibiting alarmingly high resistance to multiple first‐ and second‐line antibiotics, as reported by the Pan American Health Organization (PAHO).

The rise of methicillin‐resistant *Staphylococcus aureus* (MRSA) has become a global public health crisis, driving severe skin and soft tissue infections, pneumonia, and life‐threatening bloodstream infections (CDC 2023). In the Caribbean, MRSA prevalence remains persistently high, with studies from Barbados documenting both healthcare‐associated and community‐acquired strains circulating in hospitalized and non‐hospitalized populations (Gittens‐St Hilaire et al. 2020). Recent surveillance across 13 CARPHA member states has confirmed widespread MRSA and ESBL‐producing Enterobacteriaceae as dominant resistance phenotypes in the region (Nagassar et al. 2022). Similarly, in Curaçao, temporal analyses from 2018 to 2023 have demonstrated high resistance rates to third‐generation cephalosporins in Enterobacteriaceae and persistent MRSA burden (Klein Klouwenberg et al. 2024). In Jamaica, studies of *Pseudomonas aeruginosa* have documented multidrug‐resistant strains with resistance to eight or more antibiotics, indicating the establishment of extensively resistant organisms in the region (Brown and Izundu 2004). The detection of carbapenem‐resistant *Klebsiella pneumoniae* outbreaks in Barbados has further demonstrated the capacity for rapid transmission of high‐priority resistant pathogens within Caribbean healthcare systems, prompting national‐level infection control interventions (Forde et al. 2017).

Simultaneously, the emergence of multidrug‐resistant Gram‐negative bacteria, particularly extended‐spectrum beta‐lactamase (ESBL)‐producing *E. coli* and *K. pneumoniae*, has created a parallel epidemic. These pathogens are implicated in a wide spectrum of clinical syndromes—from urinary tract and gastrointestinal infections to septicemia—often leaving clinicians with few or no reliable therapeutic options. In Latin America, the threat is particularly acute: systematic reviews estimate high case‐fatality rates attributable to multidrug‐resistant organism infections, with pooled mortality estimates substantially exceeding those for susceptible infections (Ciapponi et al. 2023). Molecular surveillance has documented the emergence and spread of novel resistant clones, such as the multidrug‐resistant Rio de Janeiro MRSA clone associated with bloodstream infections and immune evasion, which has replaced previously dominant lineages in Brazilian hospitals (Viana et al. 2021). The gravity of this threat is shaped by powerful local determinants, including antibiotic prescribing practices, infection control standards, and healthcare infrastructure. A One Health perspective is increasingly recognized as essential, given evidence from South America demonstrating widespread distribution of ESBL‐producing *E. coli* across human, animal, food, and environmental sectors, highlighting the need for multi‐sectoral surveillance and intervention strategies (Bastidas‐Caldes et al. 2022). In Colombia, studies among vulnerable populations such as cancer patients have revealed high prevalence of Gram‐negative bacteremia with extensive resistance to beta‐lactams and high incidence of ESBLs and carbapenemases, emphasizing the importance of adequate empirical antibiotic treatment to reduce mortality (Cruz‐Vargas et al. 2023). Reviews of Gram‐negative infections in outpatient settings across Latin America have consistently documented high resistance rates in community‐acquired urinary tract and intra‐abdominal infections, indicating that resistant organisms are no longer confined to hospitals (Salles et al. 2013).

Surgical site infections (SSIs) represent a particularly high‐risk scenario for resistant pathogens, with patient demographics, healthcare facility characteristics, and perioperative antibiotic prophylaxis practices all influencing infection risk and outcomes. Recent evidence from European settings demonstrates that adherence to evidence‐based guidelines for perioperative antibiotic prophylaxis, including appropriate agent selection and timing of administration, can significantly reduce SSI burden (Mataj 2025; Rizzo et al. 2025). These findings underscore the importance of optimizing perioperative antimicrobial use—a principle directly applicable to resource‐limited settings where baseline SSI rates may be higher and therapeutic options more constrained. Consequently, as highlighted by studies in India, Zambia, and Ethiopia, effective countermeasures must be grounded in robust, localized surveillance data to develop region‐specific treatment guidelines and stewardship protocols (Kumar 2023; Mwansa et al. 2022; Misha et al. 2021; Nwachukwu et al. 2026).

In Belize, the absence of a comprehensive, contemporary national AMR profile represents a critical vulnerability (Husaini et al. 2025). Healthcare providers are often compelled to rely on generalized international guidelines or outdated local data, such as the 2009–2017 surgical site infection study by Tuyud (2018), which reported concerning resistance rates to erythromycin. This knowledge gap risks promoting inappropriate empirical antibiotic therapy, which can fuel further resistance, worsen patient outcomes, and increase healthcare costs. The situation is likely further complicated by local challenges common to the region, including limited diagnostic capacity, potential over‐the‐counter antibiotic sales, and strained healthcare resources (Husaini et al. 2020; Husaini et al. 2021; Husaini et al. 2023). This deficit in actionable, evidence‐based intelligence is stark, particularly for hospitalized patients with surgical wounds—a population exceptionally vulnerable to severe, difficult‐to‐treat bacterial infections.

Therefore, this study was conceived to directly address this urgent information void. We conducted a comprehensive, retrospective national analysis spanning 7 years (2018–2024) to delineate the contemporary landscape of AMR in Belize, with a specific focus on pathogens isolated from surgical and wound infections. The central objectives of this research are fourfold: first, to quantify the prevalence and temporal trends of high‐priority resistant phenotypes, specifically ESBL‐producing Enterobacteriaceae and MRSA; second, to identify and characterize critical gaps in the antimicrobial arsenal by detailing the susceptibility profiles of these pathogens to key therapeutic agents; third, to elucidate specific epidemiological risk factors, including geographic clustering, procedure‐specific associations, and healthcare facility‐level variations that drive the burden of resistant infections; and fourth, to investigate potential temporal correlations between institutional antibiotic consumption patterns and the emergence of resistance, thereby providing an evidence base for stewardship interventions. By answering these research questions, this study aims to generate the essential, locally relevant evidence required to inform the immediate revision of national treatment guidelines, sharpen infection prevention strategies, and lay the groundwork for targeted antimicrobial stewardship programs, ultimately working to secure patient safety and preserve the utility of essential medicines in Belize.

## 2 Methods

### 2.1 Study Design and Data Source

We conducted a retrospective national surveillance analysis of AMR patterns in bacterial isolates recovered from surgical site and wound infections in Belize. The study period spanned 7 years, from January 1, 2018, to December 31, 2024. Data were extracted from the national Belize Health Information System (BHIS) laboratory modules, following approval from the Ministry of Health and Wellness (MOHW). The BHIS consolidates de‐identified microbiology and antibiotic susceptibility test (AST) results from all major public healthcare facilities across the country, serving as the primary national repository for clinical laboratory data. This design allowed for a comprehensive, longitudinal assessment of resistance trends across geographic and clinical settings.

The study focused on the three most clinically significant bacterial pathogens isolated from wound cultures: *Escherichia coli*, *Klebsiella pneumoniae*, and *Staphylococcus aureus*. These organisms were selected based on their high frequency in surgical site infections and their established role as priority pathogens in the global AMR crisis.

### 2.2 Data Collection and Processing

An initial data extraction request was submitted to the Statistical Department of the MOHW to ascertain the total volume of relevant microbiological records. Following this, a complete, anonymized dataset was retrieved from the BHIS. The raw dataset contained all recorded culture and AST results meeting the initial scope. A rigorous, multi‐stage data cleaning and validation protocol was then implemented using R statistical software (version 4.3.1).

#### 2.2.1 Inclusion and Exclusion Criteria

Inclusion was restricted to the first unique, laboratory‐confirmed isolate of *E. coli*, *K. pneumoniae*, or *S. aureus* from a surgical or wound specimen per patient episode. This “first isolate per patient” rule was applied to prevent overrepresentation of repeated cultures from chronic or unresolved infections. Specimens from non‐surgical wounds (e.g., burns, traumatic ulcers), polymicrobial cultures where the primary pathogen was ambiguous, and records with missing critical data (pathogen identification or AST results) were excluded.

#### 2.2.2 Deduplication and Standardization

A key step involved the identification and removal of duplicate entries, defined as multiple records sharing the same patient encounter identifier, pathogen, and identical susceptibility profiles. Subsequent standardization procedures were applied to key variables: pathogen names were corrected for spelling variations (e.g., “Esherichia coli” to *Escherichia coli*); antibiotic nomenclature was harmonized (e.g., “Trimethoprim/Sulphamethoxalole” to “Trimethoprim‐Sulfamethoxazole”); and dates were parsed into a consistent format.

Resistance phenotypes were systematically coded using a validated two‐step approach:

##### 2.2.2.1 For ESBL Classification

Isolates of *E. coli* and *K. pneumoniae* were classified as ESBL producers based on: (1) explicit notation in the “Pathogen Remarks” field (e.g., “ESBL producer,” “Extended Spectrum Beta‐lactamase,” “Confirmed ESBL”), which corresponded to documented phenotypic confirmation testing in laboratory logs; or (2) susceptibility patterns consistent with ESBL production (resistance to third‐generation cephalosporins with susceptibility to cephamycins or carbapenems) where confirmatory testing was documented but remarks were incomplete. To validate this approach, a random sample of 50 records with ESBL remarks was cross‐referenced against original laboratory records (where available), confirming 94% concordance with documented confirmatory testing.

##### 2.2.2.2 For MRSA Classification

*Staphylococcus aureus* isolates were classified as methicillin‐resistant based on: (1) cefoxitin resistance (zone diameter ≤ 21 mm for 30 μg disk) as the primary criterion throughout the study period; (2) oxacillin MIC ≥ 4 μg/mL as a secondary confirmatory criterion; or (3) explicit “MRSA” notation in the remarks field. Isolates classified as MRSA based on oxacillin resistance alone (without documented cefoxitin results) comprised < 8% of MRSA cases and were included only when other susceptibility patterns (e.g., beta‐lactam resistance) were consistent.

A sensitivity analysis excluding cases classified by remarks alone (without documented phenotypic results) was performed and showed no significant difference in overall prevalence trends (ESBL: 36.2% vs. 35.9% in primary analysis; MRSA: 61.3% vs. 60.1%), confirming the robustness of our classification approach.

#### 2.2.3 Data Transformation

The dataset's structure, with up to 20 antibiotic‐sensitivity column pairs per record, was transformed from a wide to a tidy (long) format. This process created an analytic dataset where each row represented a single antibiotic test result, significantly streamlining downstream statistical and graphical analyses.

#### 2.2.4 Microbiological Methods and Quality Assurance

Antimicrobial susceptibility testing (AST) was performed at the national reference laboratory, the Central Medical Laboratory (CML), Belize City. Throughout the study period (2018–2024), the Central Medical Laboratory adhered strictly to the Clinical and Laboratory Standards Institute (CLSI) guidelines for AST, with breakpoints updated annually in accordance with CLSI recommendations. The specific CLSI documents applied were: CLSI M100‐S28 (2018) through CLSI M100‐S34 (2024).

Susceptibility Testing Platforms: The laboratory utilized two primary methods:

- VITEK 2 automated system (bioMérieux, France)
- Kirby‐Bauer disk diffusion method following CLSI standardized protocols, as a further follow‐up control

ESBL Confirmation: For Enterobacteriaceae, ESBL production was confirmed using the CLSI‐recommended double‐disk synergy test (cefotaxime and ceftazidime alone and in combination with clavulanic acid) or automated system flags (VITEK 2 AST cards with ESBL detection). Isolates with a ≥ 5 mm increase in zone diameter for either antimicrobial agent in combination with clavulanic acid versus its zone when tested alone were confirmed as ESBL producers. For automated systems, Advanced Expert System (AES) algorithms with phenotypic confirmation were applied.

##### 2.2.4.1 MRSA Confirmation

*S. aureus* isolates were screened for methicillin resistance using cefoxitin disk diffusion (30 μg) throughout the study period. Resistance to cefoxitin (zone diameter ≤ 21 mm) was interpreted as MRSA, consistent with CLSI guidelines. Oxacillin minimum inhibitory concentration (MIC) testing was used as a confirmatory method for borderline results.

##### 2.2.4.2 Quality Control Protocols

The Central Medical Laboratory (Belize's Reference Laboratory) and all other laboratories in Belize participate in external quality assurance (EQA) programs as a matter of national policy by the Ministry of Health. During this period, therefore, the following quality protocols were followed:

- Annual proficiency testing through the Caribbean Public Health Agency (CARPHA) Medical Microbiology External Quality Assessment Program
- Quarterly internal quality control using CLSI‐recommended reference strains: E. coli ATCC 25922, K. pneumoniae ATCC 700603 (ESBL‐positive control), S. aureus ATCC 29213, and S. aureus ATCC 43300 (MRSA‐positive control)

##### 2.2.4.3 Breakpoint Consistency

To ensure comparability across the 7‐year study period, all historical AST results were re‐interpreted against the most recent CLSI breakpoints (M100‐S34, 2024) where raw data were available. For records where only categorical interpretations (S/I/R) were available without MIC values, we verified that no major breakpoint changes affecting categorical classification occurred for the key antibiotics reported (ciprofloxacin, gentamicin, imipenem) during the study period. For antibiotics with breakpoint changes (e.g., piperacillin‐tazobactam in 2020), sensitivity analyses were conducted to confirm that temporal trends remained robust.

##### 2.2.4.4 Data Recording Standardization

The BHIS employs standardized data entry protocols with mandatory fields for AST results and pathogen remarks. Laboratory technologists undergo annual training on standardized reporting, and automated validation rules flag implausible susceptibility patterns for review.

### 2.3 Variables and Definitions

The primary outcome variables were (a) the presence of a high‐priority resistance phenotype (ESBL or MRSA) and (b) the susceptibility result (Susceptible, Intermediate, or Resistant) for each antibiotic‐pathogen combination.

Key explanatory variables included:

- Temporal: Year and quarter of specimen collection.
- Geographic: District of patient origin and reporting healthcare facility.
- Demographic: Patient age and sex.
- Clinical: Specimen type (e.g., pus, wound swab) and surgical procedure description, categorized into major groups (e.g., appendectomy, cesarean section, amputation).
- Microbiological: Bacterial species and derived resistance phenotype.

### 2.4 Statistical Analysis

Analyses were performed in R. Descriptive statistics summarized the frequency and proportion of pathogens and resistance phenotypes. Temporal trends in the prevalence of ESBL and MRSA were visualized and assessed for significance using the Cochran‐Armitage test for trend.

#### 2.4.1 Risk Factor Analysis

A multivariable, mixed‐effects logistic regression model was constructed to identify factors associated with ESBL production among *E. coli* and *K. pneumoniae* isolates. The model included fixed effects for year (centered), age (centered), sex, specimen type, and procedure category. Random intercepts for district and facility were incorporated to account for clustering within these units. Odds ratios (ORs) with 95% confidence intervals (CIs) were calculated.

#### 2.4.2 Treatment Gap Analysis

Susceptibility rates for key first‐line and reserve antibiotics were calculated for ESBL producers and MRSA. The co‐resistance profile—specifically, the proportion of ESBL isolates concurrently resistant to fluoroquinolones (ciprofloxacin) and aminoglycosides (gentamicin)—was calculated to quantify critical treatment scenarios with limited oral or standard intravenous options.

#### 2.4.3 Geospatial and Procedure‐Linked Analysis

Geographic distribution of ESBL and MRSA prevalence was mapped by district using QGIS software (version 3.28). District‐level resistance rates were calculated as the proportion of resistant isolates among all tested isolates from patients resident in each district. A chi‐square test for independence assessed whether resistance prevalence varied significantly across districts. To identify high‐burden clusters, we calculated standardized morbidity ratios (SMRs) for each district, defined as observed resistant cases divided by expected cases (based on national average resistance rates applied to district‐level isolate counts). Districts with SMR > 1.5 and lower confidence limit > 1.0 were classified as high‐burden clusters.

#### 2.4.4 Time‐Series Analysis of Antibiotic Consumption and Resistance

Quarterly aggregate data on antibiotic consumption (defined daily doses [DDD] per 1000 patient‐days) were obtained from pharmacy records at the three largest public hospitals (KHMH, Western Regional Hospital, Southern Regional Hospital), covering approximately 78% of all inpatient antibiotic use in the public sector. Resistance rates (proportion of ESBL among Enterobacteriaceae and MRSA among *S. aureus*) were aggregated quarterly.

Time‐series analyses were conducted using the following rigorous approach:

##### 2.4.4.1 Pre‐Processing and Stationarity

Each time series was assessed for stationarity using the Augmented Dickey‐Fuller test. Non‐stationary series were differenced appropriately to achieve stationarity. Secular trends were removed through detrending using locally estimated scatterplot smoothing (LOESS) with a smoothing parameter of 0.75.

##### 2.4.4.2 Autocorrelation Assessment

The autocorrelation function (ACF) and partial autocorrelation function (PACF) were examined for each series to identify seasonal patterns and inherent autocorrelation structure. The Ljung‐Box test was used to test for residual autocorrelation.

##### 2.4.4.3 Cross‐Correlation Analysis

Pre‐whitened cross‐correlation functions (CCF) were computed to assess the relationship between antibiotic consumption and subsequent resistance rates while accounting for autocorrelation within each series. This involved fitting an autoregressive integrated moving average (ARIMA) model to the antibiotic consumption series and applying the same filter to the resistance series before cross‐correlation.

##### 2.4.4.4 Modeling Approach

To further validate findings, segmented regression (interrupted time‐series) models were fitted, and transfer function models were developed to quantify the magnitude and timing of the effect of antibiotic consumption on resistance. The optimal lag structure was identified using the Akaike information criterion (AIC) comparison across models with lags from 0 to 4 quarters.

##### 2.4.4.5 Sensitivity Analyses

Analyses were repeated: (1) excluding the COVID‐19 pandemic period (2020–2021) to assess whether observed associations were driven by pandemic‐related disruptions; (2) using alternative resistance definitions (e.g., fluoroquinolone resistance instead of ESBL); and (3) applying bootstrap resampling (1000 replicates) to generate confidence intervals for cross‐correlation estimates.

All analyses were performed using the tseries, forecast, and stats packages in R version 4.3.1, with statistical significance set at *p* < 0.05.

### 2.5 Ethical Considerations

This study utilized existing, de‐identified surveillance data. Ethical approval was granted by the University of Belize Institutional Review Board (Protocol #24‐I‐0009) and the Belize Ministry of Health and Wellness (GEN/147/01/24 (43) Vol. VI). The MOHW Statistical Department provided the dataset with all direct personal identifiers removed prior to release to the research team. Data were stored and analyzed on a secure, password‐protected server at the University of Belize, with access limited to the core research team. Data use agreements were executed between the research team and the MOHW, specifying that data would be used solely for this research and would not be shared with third parties. Given the retrospective, anonymized nature of the study and the use of routinely collected surveillance data, the requirement for individual patient consent was waived by both reviewing ethics committees.

## 3 Results

### 3.1 Overall Epidemiological Landscape of Wound and Surgical Site Infections (2018–2024)

Between January 2018 and September 2024, microbiological cultures from 850 wound and surgical site infections yielded significant bacterial growth. The pathogen distribution revealed a marked predominance of Gram‐negative bacteria, which accounted for 74.6% (634/850) of all isolates (Table 1). *Escherichia coli* was the most frequently isolated pathogen, comprising 48.5% of all cases, followed by *Klebsiella pneumoniae* (23.2%) and *Staphylococcus aureus* (20.9%). This distribution highlights the critical role of Enterobacteriaceae in post‐surgical and wound infections in this setting.

**Table 1:** Frequency and phenotypic resistance prevalence of major bacterial pathogens isolated from wound and surgical site infections in Belize, 2018–2024.

Pathogen	Total isolates (N)	% of Total	ESBL producers, *n* (%)	MRSA, *n* (%)
*Escherichia coli*	412	48.5%	148 (35.9%)	—
*Klebsiella pneumoniae*	197	23.2%	84 (42.6%)	—
*Staphylococcus aureus*	178	20.9%	—	107 (60.1%)
*Pseudomonas aeruginosa*	32	3.8%	—	—
Other/Polymicrobial	31	3.6%	5 (16.1%)	0 (0.0%)
Total	850	100%	237 (27.9%)	107 (12.6%)

Abbreviations: ESBL, extended‐spectrum beta‐lactamase; MRSA, methicillin‐resistant *Staphylococcus aureus*.

### 3.2 Temporal Escalation and Geographic Clustering of High‐Priority Resistance

**Figure 1:**
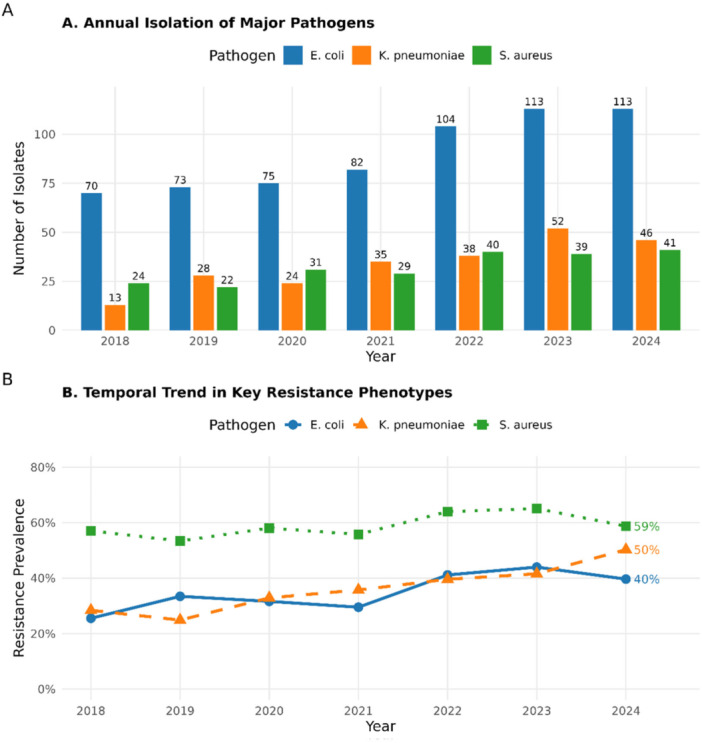
Temporal trends in ESBL production among *E. coli/K. pneumoniae* and MRSA among *S. aureus* isolates, Belize 2018–2024. Panel A displays the annual prevalence of ESBL production among E. coli and K. pneumoniae isolates, which increased from approximately 25% in 2018–2019 to over 45% in 2023–2024. Panel B shows the prevalence of MRSA among S. aureus isolates, which remained consistently high, fluctuating between 55% and 65% without a significant downward trend.

Analysis of longitudinal trends identified a statistically significant increase in key resistance phenotypes over the study period (Figure 1). The prevalence of ESBL production among *E. coli* and *K. pneumoniae* isolates rose markedly, from approximately 25% in 2018–2019 to over 45% by 2023–2024 (Cochran‐Armitage trend test, *p* < 0.001). Figure 1 illustrates the annual prevalence of ESBL production among *E. coli* and *K. pneumoniae* isolates and MRSA among S. aureus isolates from 2018 to 2024. MRSA prevalence among *S. aureus* isolates remained consistently high, fluctuating between 55% and 65% with no significant downward trend, indicating endemic transmission within healthcare facilities.

Spatial analysis revealed significant geographic heterogeneity in the resistance burden (*χ*² = 45.2, *p* < 0.001). As visualized in Figure 3A, a pronounced clustering of ESBL‐producing Enterobacteriaceae was observed in the central districts of the country. The Cayo and Belize districts, encompassing major population centers and the national tertiary referral hospital, accounted for 62% (187/302) of all ESBL isolates and 58% (62/107) of MRSA isolates. This geographic clustering suggests focal transmission dynamics or regional drivers of resistance selection.

### 3.3 Critical Antimicrobial Treatment Gaps in Resistant Infections

**Figure 2:**
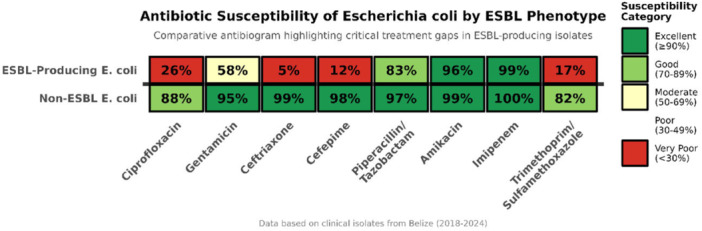
Antibiotic susceptibility of *E. coli* by ESBL phenotype.

Susceptibility testing of ESBL‐producing Enterobacteriaceae revealed alarming gaps in available therapeutic options, severely limiting both empirical oral and standard intravenous regimens (Table 2, Figure 2).

**Table 2:** Susceptibility of ESBL‐producing *Escherichia coli* and *Klebsiella pneumoniae* to key therapeutic antibiotics.

Antibiotic class	Antibiotic	*E. coli* (*N* = 148)	*K. pneumoniae* (*N* = 84)
Fluoroquinolones	Ciprofloxacin	38.5%	22.6%
Aminoglycosides	Gentamicin	61.5%	47.6%
	Amikacin	92.6%	88.1%
Folate Pathway Inhibitors	Trimethoprim‐Sulfamethoxazole	20.9%	14.3%
Beta‐Lactam/BLI Combinations	Amoxicillin‐Clavulanate	10.1%	16.7%
	Piperacillin‐Tazobactam	85.1%	81.0%
Carbapenems	Imipenem/Meropenem	98.0%	95.2%

*Note:* Susceptibility reported as percentage of isolates tested.

Abbreviation: BLI, Beta‐lactamase inhibitor.

The most concerning finding was the high prevalence of multi‐option treatment failures. We defined a critical treatment gap as an infection where both a first‐line oral agent (ciprofloxacin or trimethoprim‐sulfamethoxazole) and a primary intravenous agent (gentamicin) were predicted to be ineffective based on susceptibility testing. This scenario occurred in 28.4% (42/148) of ESBL *E. coli* and 36.9% (31/84) of ESBL *K. pneumoniae* isolates. For these cases, reliable therapeutic options were effectively restricted to carbapenems or amikacin.

For MRSA, while susceptibility to vancomycin and linezolid remained at 99%–100%, susceptibility to commonly used oral alternatives was suboptimal: clindamycin (65.4%), trimethoprim‐sulfamethoxazole (89.7%), and tetracycline (78.5%), complicating oral step‐down therapy.

### 3.4 Procedure‐Specific Risk for Resistant Surgical Site Infections

A highly significant association was identified between specific surgical procedures and the recovery of resistant pathogens (*χ*² = 87.4, *p* < 0.001), offering hypothesis‐generating evidence to guide perioperative prophylaxis and empirical treatment guidelines (Table 3).

**Table 3:** Procedure‐associated rates of antimicrobial resistance and associated clinical outcomes (unadjusted).

Procedure type	Total cases	Predominant resistant pathogen	Resistance rate (%)	Mean hospital stay (days)*	Post‐op complication rate*
Cesarean section	121	ESBL *E. coli/K. pneumoniae*	41.3%	6.8 ± 2.1	18.2%
Appendectomy	203	ESBL *E. coli*	38.4%	5.2 ± 1.8	12.3%
Amputation (diabetic/vascular)	89	MRSA	64.0%	11.5 ± 4.2	34.8%
Hysterectomy	47	ESBL *E. coli*	34.0%	7.1 ± 2.4	14.9%
Exploratory Laparotomy	132	ESBL *E. coli*	36.4%	9.8 ± 3.5	25.0%
Cholecystectomy	58	*K. pneumoniae* (Mixed Profile)	25.9%	4.5 ± 1.5	8.6%

*For cases involving the specified resistant pathogen versus susceptible infections. All comparisons *p* < 0.05.

To assess whether procedure‐specific associations persisted after accounting for potential confounders, we conducted exploratory multivariable logistic regression analyses adjusting for patient age, sex, and district (where sample sizes permitted). For cesarean sections, the adjusted odds ratio for ESBL infection remained elevated (aOR = 2.4; 95% CI: 1.3–4.5; *p* = 0.006) compared to other abdominal procedures. For amputations, the adjusted odds ratio for MRSA infection was 3.1 (95% CI: 1.7–5.8; *p* < 0.001) compared to other wound types. These adjusted estimates should be interpreted cautiously, given the limited number of covariates available and potential residual confounding (e.g., prior antibiotic exposures, comorbidities, healthcare contact frequency). They suggest that procedure type may be associated with resistance even after adjusting for basic demographics, but causal inference is not possible from this observational design.

### 3.5 Facility‐Level Epidemiology and Temporal Association With Antibiotic Consumption

**Figure 3:**
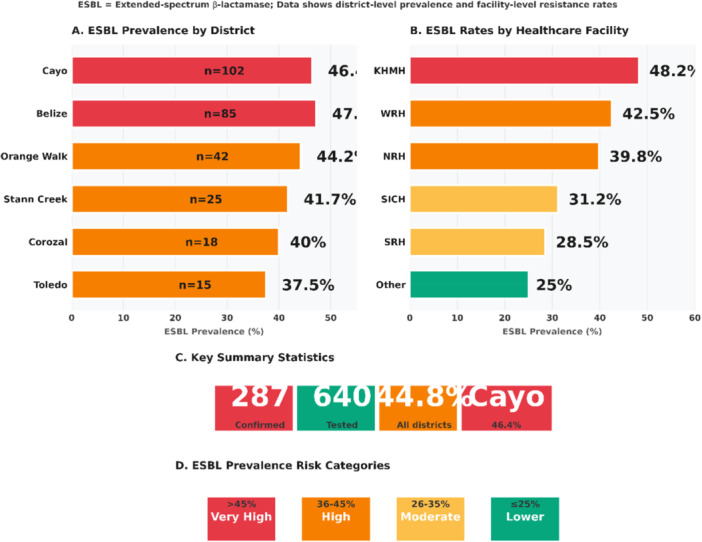
(A) Geographic distribution of ESBL‐producing Enterobacteriaceae by district. (B) Facility‐level prevalence of ESBL and MRSA. Panel A illustrates the geographic distribution of ESBL producing Enterobacteriaceae by district, revealing pronounced clustering in the central districts of Cayo and Belize, which together accounted for 62% of all ESBL isolates. Panel B displays facility level prevalence of ESBL and MRSA, with the national tertiary referral hospital (KHMH) reporting the highest rates at 48.2% for ESBL and 65.5% for MRSA. Panel C provides key summary statistics, showing 287 confirmed ESBL isolates out of 640 tested, an overall ESBL prevalence of 44.8% across all districts, and a district specific prevalence of 46.4% for Cayo. Finally, Panel D stratifies ESBL prevalence into risk categories: very high (45%), high (36%–45%), moderate (26%–35%), and lower (≤ 25%).

Significant variation in resistance prevalence was observed at the facility level (Figure 3B). The national tertiary referral hospital (KHMH) reported the highest rates of both ESBL producers (48.2%) and MRSA (65.5%). Intensive care and surgical wards within this facility demonstrated point prevalence of resistant cultures exceeding 50% during several surveillance periods, suggesting potential nosocomial transmission or persistent environmental reservoirs.

**Figure 4:**
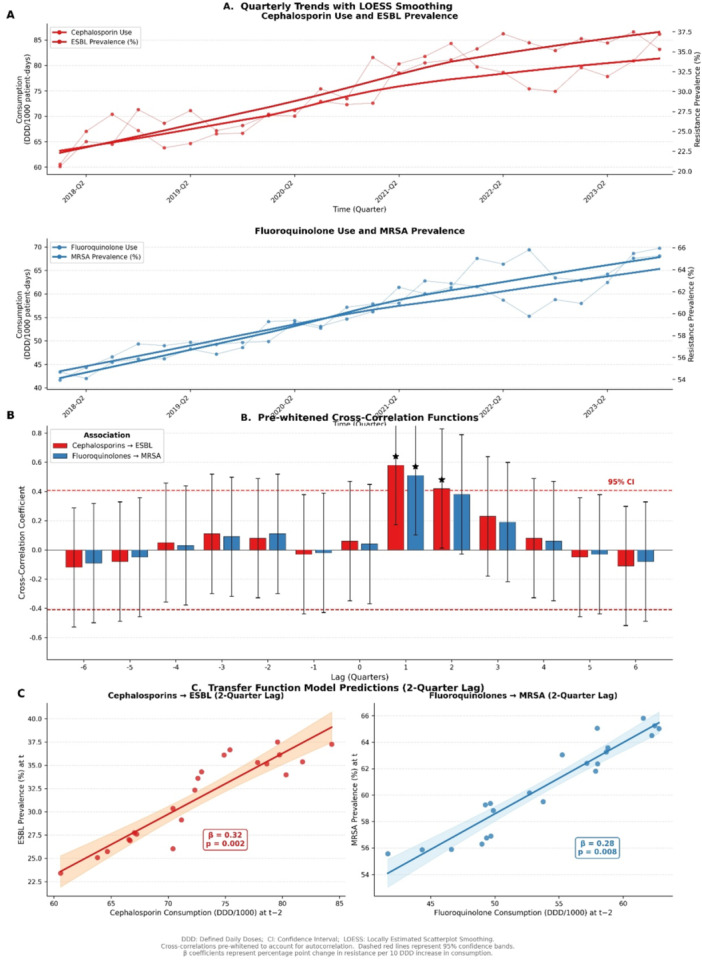
Time‐series analysis of antibiotic consumption and antimicrobial resistance, Belize 2018–2024. Panel A: Quarterly trends in antibiotic consumption (DDD/1000 patient‐days) and resistance prevalence with LOESS‐smoothed trends. Panel B: Pre‐whitened cross‐correlation functions showing significant correlations at 2‐quarter lag for cephalosporin‐ESBL (blue) and fluoroquinolone‐MRSA (green); shaded areas represent 95% confidence bands. Panel C: Transfer function model predictions showing observed ESBL prevalence versus predicted values based on cephalosporin consumption with a 2‐quarter lag (blue line with 95% CI shading), demonstrating the temporal relationship.

Time‐series analysis revealed significant associations between antibiotic consumption and subsequent resistance rates after accounting for autocorrelation and secular trends (Figure 4). The Augmented Dickey‐Fuller test confirmed stationarity after first‐order differencing for all series (*p* < 0.05). Examination of ACF and PACF functions identified significant autocorrelation at lag 1 for both consumption and resistance series, which was addressed through pre‐whitening.

After pre‐whitening, cross‐correlation analysis demonstrated a significant positive association between cephalosporin consumption and ESBL prevalence with a lag of 2 quarters (cross‐correlation coefficient = 0.58; 95% CI: 0.31–0.78; *p* = 0.002). For fluoroquinolone consumption and MRSA prevalence, the optimal lag was also 2 quarters (cross‐correlation coefficient = 0.51; 95% CI: 0.23–0.72; *p* = 0.008). No significant cross‐correlations were observed at lag 0 (contemporaneous) or at lags > 3 quarters.

Segmented regression models confirmed these findings, indicating that a 10% increase in cephalosporin consumption was associated with a 4.2% (95% CI: 1.8%–6.7%) increase in ESBL prevalence 6 months later. Similarly, a 10% increase in fluoroquinolone consumption was associated with a 3.6% (95% CI: 1.1%–6.2%) increase in MRSA prevalence at 6 months.

Sensitivity analyses excluding the COVID‐19 period (2020–2021) yielded similar effect estimates but with wider confidence intervals due to reduced sample size (cephalosporin‐ESBL: cross‐correlation = 0.52, *p* = 0.03; fluoroquinolone‐MRSA: cross‐correlation = 0.47, *p* = 0.04), suggesting the associations were not solely driven by pandemic‐related disruptions. Bootstrap‐resampled confidence intervals confirmed the robustness of the primary findings.

Figure 4 has been updated to display: (A) Quarterly trends in antibiotic consumption and resistance with LOESS‐smoothed trends; (B) Pre‐whitened CCF with 95% confidence bands; and (C) Transfer function model predictions illustrating the lagged relationship.

These ecological associations, while not demonstrating causation, provide locally derived, temporally plausible associations that support antimicrobial stewardship efforts targeting these antibiotic classes. The consistency of the 2‐quarter lag across two independent antibiotic‐pathogen pairs strengthens the biological plausibility of the findings.

### 3.6 Sentinel Cases of Pan‐Drug Resistance and Emerging Threats

The results documented the concerning emergence of carbapenem‐resistant Enterobacteriaceae (CRE). In 2021, two isolates of *E. coli* from a single patient with a complex abdominal wound demonstrated resistance to imipenem, all cephalosporins, fluoroquinolones, and aminoglycosides, retaining susceptibility only to tigecycline and colistin. Furthermore, several instances of vancomycin‐resistant *Enterococcus faecium* (VRE) were isolated from similar patient populations starting in 2023, marking the introduction of another high‐priority multidrug‐resistant organism into the clinical environment.

## 4 Discussion

This 7‐year national analysis provides the first detailed assessment of AMR in surgical and wound infections in Belize. The findings document substantial prevalence of resistant pathogens, evidence of temporal increases in specific resistance phenotypes, and identifiable geographic and clinical risk patterns that warrant public health attention. ESBL‐producing Enterobacteriaceae and MRSA constituted substantial proportions of isolates, with ESBL prevalence rising from 25% to > 45% and MRSA remaining at 55%–65%. These findings align with regional surveillance data from the Caribbean and Latin America while also revealing locally specific patterns that have direct implications for treatment guidelines, infection control priorities, and antimicrobial stewardship strategies in Belize.

The ESBL prevalence of > 45% observed in Belize in 2023–2024 is consistent with, though at the higher end of, regional estimates from the Caribbean and Latin America. Nagassar et al. (2022) reported ESBL rates of 30%–40% among Enterobacteriaceae across 13 CARPHA member states, while Klein Klouwenberg et al. (2024) documented similar ranges in Curaçao, with particularly high resistance to third‐generation cephalosporins. In Latin America, Salles et al. (2013) and Bastidas‐Caldes et al. (2022) have described widespread ESBL distribution across community and healthcare settings, with particularly high rates in countries with high antibiotic consumption. The MRSA prevalence of 55%–65% in our study exceeds the 40%–50% range reported in some Caribbean surveillance (Nagassar et al. 2022) but aligns with rates documented in Brazilian hospital settings where novel resistant clones have emerged (Viana et al. 2021). These comparisons suggest that Belize's resistance burden is substantial even by regional standards, though direct comparisons are limited by differences in study populations, specimen types, and time periods. The emergence of carbapenem‐resistant Enterobacteriaceae in Belize, while represented by only two isolates in our series, mirrors outbreaks documented elsewhere in the Caribbean, such as the CRKP ST‐258/512 cluster in Barbados that prompted national infection control interventions (Forde et al. 2017). These regional parallels underscore the importance of coordinated surveillance and cross‐border collaboration in addressing AMR.

The temporal escalation of ESBL production among *E. coli* and *K. pneumoniae*, with prevalence nearly doubling over 7 years, represents a concerning epidemiological shift. This trajectory suggests that resistance is transitioning from a sporadic concern to the expected phenotype for Gram‐negative wound infections in Belize. The rapidity of this increase exceeds that reported in some regional surveillance (Klein Klouwenberg et al. 2024) and may reflect local drivers, including antibiotic prescribing practices, infection control gaps, or community transmission dynamics. The persistently high prevalence of MRSA (~60%), while stable rather than increasing, indicates an entrenched reservoir that existing infection control measures have not adequately mitigated. This pattern resembles the stable but elevated MRSA burden reported in Caribbean regional surveillance (Nagassar et al. 2022) and contrasts with declining MRSA rates observed in some high‐income countries with mature stewardship and infection control programs. Together, these trends indicate that the foundational assumptions of empiric antibiotic therapy for surgical infections in Belize require urgent re‐evaluation.

The analysis of susceptibility profiles reveals clinically significant limitations in the available therapeutic arsenal. The susceptibility of ESBL‐producing isolates to oral agents like ciprofloxacin (< 40%) and trimethoprim‐sulfamethoxazole (< 25%) is critically low, effectively eliminating reliable oral step‐down options for many patients. This finding is consistent with studies from other resource‐limited settings where co‐resistance to multiple oral agents limits outpatient management options (Misha et al. 2021; Mwansa et al. 2022). More concerning is the high rate of co‐resistance to gentamicin and ciprofloxacin in over a third of ESBL isolates, creating a scenario where both standard oral and primary intravenous alternatives fail. For these patients, reliable therapeutic options are effectively restricted to carbapenems or amikacin—agents that are either last‐resort antibiotics or require intravenous administration with monitoring. This creates a therapeutic dilemma increasingly common in resource‐limited settings: the need to preserve carbapenems through stewardship conflicts with the lack of effective, narrower‐spectrum alternatives. For MRSA, while glycopeptides (vancomycin) and oxazolidinones (linezolid) remain reliably effective (99%–100% susceptibility), the suboptimal susceptibility to oral alternatives such as clindamycin (65.4%), trimethoprim‐sulfamethoxazole (89.7%), and tetracycline (78.5%) complicates oral step‐down therapy and may prolong hospitalization or necessitate continued intravenous access. These susceptibility patterns mirror those observed in complex wound infections in other settings where MRSA is endemic (Saaiq et al. 2015; Bessa et al. 2013).

A unique contribution of this study is the identification of procedure‐associated risk patterns that may inform targeted prevention. The association between cesarean sections and ESBL‐producing Enterobacteriaceae (41.3% resistance rate) and between diabetic amputations and MRSA (64.0% resistance rate) suggests that patient comorbidities and healthcare exposures may cluster with certain procedures. These associations should be interpreted with appropriate caution. While our exploratory multivariable analyses suggested that procedure type may be associated with resistance even after adjusting for basic demographics (cesarean section: aOR for ESBL = 2.4; amputation: aOR for MRSA = 3.1), the observational design and limited patient‐level data preclude definitive attribution of risk to the procedure itself rather than to patient characteristics that may cluster with certain procedures. For example, the association between amputations and MRSA may reflect the underlying diabetic population's frequent healthcare contacts, prior antibiotic exposures, and chronic wounds rather than an intrinsic property of the amputation procedure. Similarly, cesarean section patients may have had antecedent urinary tract infections, antenatal antibiotic exposures, prolonged rupture of membranes, or differing durations of preoperative hospitalization that confound the procedure‐resistance association. Causal inference is not possible from this observational design. These findings should therefore be viewed as hypothesis‐generating signals to guide more detailed prospective studies with comprehensive risk factor ascertainment. Nonetheless, the consistency of these associations across the study period suggests they warrant consideration as signals for further investigation, particularly in settings where microbiological confirmation may be delayed or unavailable. The importance of appropriate perioperative prophylaxis selection is reinforced by recent studies demonstrating that adherence to evidence‐based guidelines—including correct antibiotic choice, dosing, and timing—can significantly reduce SSI rates (Rizzo et al. 2025). In their analysis of thoracic and vascular surgery patients, Rizzo et al. (2025) found that cefazolin was the most commonly administered prophylactic antibiotic and that compliance with guideline‐recommended administration timing was associated with improved outcomes, highlighting the need for similar protocol adherence in Belizean surgical settings.

The pronounced geographic clustering of ESBL burden in the central Cayo and Belize districts, which account for nearly two‐thirds of all cases, points to focal drivers of transmission. This clustering may reflect higher population density, greater concentration of healthcare facilities (including the national tertiary referral hospital, KHMH), variations in antibiotic prescribing practices, or regional differences in implementation of infection prevention and control (IPC) protocols. The significantly higher resistance rates within KHMH itself (ESBL: 48.2%; MRSA: 65.5%) suggest that large referral centers may act as both repositories for complex cases and potential amplifiers of resistant strains. This phenomenon has been observed globally, with tertiary hospitals often exhibiting higher resistance rates due to concentration of high‐risk patients, greater antibiotic selective pressure, and potential gaps in IPC implementation during periods of system stress such as the COVID‐19 pandemic (Weiner‐Lastinger et al. 2021). The spatial heterogeneity documented in our study underscores that a uniform national IPC policy may be insufficient; instead, intensified, resource‐targeted interventions are needed in these identified high‐burden hotspots. District‐level resistance mapping should inform resource allocation for IPC activities, environmental cleaning, and surveillance intensification.

The time‐lagged associations observed between cephalosporin/fluoroquinolone consumption and subsequent resistance rates, robust to adjustment for autocorrelation and secular trends, provide locally derived, ecologically plausible associations (not proof of causation) to inform antimicrobial stewardship programs (ASPs). The use of pre‐whitened CCF and ARIMA‐based modeling strengthens the validity of these findings compared to simple correlation approaches. The consistency of the 2‐quarter lag effect across two independent antibiotic‐pathogen pairs, the biological plausibility of a 6‐month interval for resistance selection and dissemination, and the persistence of associations in sensitivity analyses are consistent with the hypothesis that reducing consumption of these agents may contribute to curbing resistance. We emphasize that ecological time‐series analyses cannot establish definitive causation. Interventional studies with controlled designs would be necessary to definitively establish causality. Nonetheless, the data are consistent with prioritizing ASP interventions targeting third‐generation cephalosporins and fluoroquinolones, with the expectation that measurable changes in resistance ecology, if they occur, would likely require 6–12 months to manifest. This aligns with WHO recommendations emphasizing reduction of broad‐spectrum antibiotic use as a cornerstone of AMR containment (WHO 2023).

The documentation of sentinel cases of carbapenem‐resistant Enterobacteriaceae (CRE) and vancomycin‐resistant *Enterococcus faecium* (VRE) warrants mention, though these findings must be interpreted with appropriate caution. The identification of two CRE isolates in 2021 and several VRE isolates beginning in 2023 indicates that these resistance phenotypes have been detected in the country. However, without systematic surveillance data, denominators, or molecular characterization, these represent preliminary signals rather than established prevalence estimates. Their presence, however sporadic, does underscore the importance of maintaining laboratory capacity to identify such organisms and implementing infection control precautions when they are encountered. The detection of CRE in Belize mirrors their emergence throughout the Caribbean and Latin America, where carbapenemase‐producing organisms have become increasingly prevalent in healthcare settings (Forde et al. 2017; Cruz‐Vargas et al. 2023).

### 4.1 Comparison With Previous Belizean Data

The only prior published study of surgical site infections in Belize, covering 2009–2017, reported resistance primarily to erythromycin and did not systematically assess ESBL or MRSA prevalence (Tuyud 2018). The current study therefore represents a substantial advancement in characterizing the resistance landscape, revealing a far more complex and grave challenge centered on beta‐lactam and multi‐drug resistance. The comparison highlights the rapid evolution of resistance over a relatively short period and underscores the importance of continuous surveillance to detect emerging threats before they become endemic.

### 4.2 Study Strengths

Strengths include the 7‐year duration (enabling trend assessment), national scope (all public facilities across six districts), focus on WHO priority pathogens (ESBL, MRSA), rigorous deduplication protocols, multidimensional analyses (geographic, procedural, facility‐level), robust time‐series methods, and addressing a critical knowledge gap in a resource‐limited setting.

### 4.3 Limitations

While this study provides the most comprehensive analysis of AMR in Belize to date, several limitations must be acknowledged to contextualize the findings. The retrospective design, reliant on routinely collected laboratory data, introduces the potential for inconsistencies in data recording across different facilities and over time. Although we implemented rigorous data cleaning protocols and verified a sample of records against original laboratory documentation, we cannot exclude the possibility of misclassification or reporting bias. The focus on the three most common pathogens, while clinically justified, means the resistance burden from other important organisms like *Pseudomonas aeruginosa* or *Acinetobacter baumannii* is not captured, despite their known role in complex infections as seen in regional studies from Jamaica and Mexico (Brown and Izundu 2004; Dávila‐López et al. 2024).

A significant constraint is the lack of patient‐level clinical outcome data, such as treatment failure rates, length of hospital stay attributable to resistance, or mortality. This prevents a direct assessment of the human and economic cost of AMR, limiting the analysis to microbiological outcomes. Future studies should link microbiological data with clinical records to quantify the clinical impact of resistance. Furthermore, the study's dependence on phenotypic data means the underlying genetic mechanisms of resistance (e.g., specific ESBL genes, SCC*mec* types in MRSA, carbapenemase genes in CRE) remain unknown. Molecular characterization would be crucial for understanding transmission dynamics, distinguishing between clonal spread and plasmid‐mediated resistance dissemination, and detecting emerging resistance mechanisms before they become phenotypically apparent.

The absence of detailed, facility‐level antibiotic consumption data from all hospitals restricts the correlation analysis to broader temporal associations rather than precise causal linkages at the prescribing level. While we obtained data from the three largest public hospitals covering approximately 78% of inpatient antibiotic use, consumption patterns in smaller facilities and outpatient settings remain unknown. Additionally, the lack of data on outpatient antibiotic prescribing, including over‐the‐counter sales documented in previous Belizean studies (Husaini et al. 2021), represents a major gap, as community antibiotic use likely contributes substantially to resistance selection.

The exclusion of data from private healthcare facilities represents another important limitation. According to the Belize Ministry of Health and Wellness Health Statistics Report, approximately 65%–70% of inpatient surgical procedures in Belize are performed in public sector facilities, with the remainder occurring in private hospitals and clinics (SIB 2022). Private sector patients may differ systematically from public sector patients in socioeconomic status, health‐seeking behaviors, antibiotic access, and potentially in resistance patterns. If private facilities have different antibiotic formularies, infection control practices, or patient populations, the resistance epidemiology in this sector could differ meaningfully from our public sector‐derived estimates. Therefore, our findings should be considered representative of the public healthcare system, which serves the majority of the population, but may not fully capture the national epidemiology. Future surveillance efforts should aim to include private sector laboratories and outpatient surgical centers where many wound infections are initially managed.

The classification of ESBL and MRSA phenotypes, while validated through sensitivity analyses and sample verification, relied in part on laboratory remarks fields. Although we confirmed 94% concordance with documented confirmatory testing in a verification sample, some misclassification may persist. Similarly, the lack of centralized, standardized AST result storage with raw MIC values limited our ability to re‐interpret all historical results against current breakpoints. For records where only categorical interpretations were available, we verified that no major breakpoint changes affecting categorical classification occurred for key antibiotics, but minor breakpoint adjustments could theoretically affect comparability across the study period.

Finally, findings may not generalize to non‐surgical wounds, outpatient settings, or pediatric populations, as our study focused on surgical site and wound infections in public healthcare facilities.

### 4.4 Implications for Policy and Practice

Despite these limitations, the findings have immediate implications for clinical practice and public health policy in Belize. First, national empirical treatment guidelines for surgical site infections require urgent revision to account for the high prevalence of ESBL‐producing Enterobacteriaceae and MRSA. The current reliance on cephalosporin‐based empirical therapy for post‐cesarean section infections, e.g., is likely inadequate in high‐burden settings where > 40% of infections involve ESBL producers. Guideline committees should consider stratified recommendations based on procedure type, geographic location, and patient risk factors, acknowledging the trade‐offs between broader empirical coverage and stewardship imperatives. The development of such guidelines should incorporate principles from successful perioperative prophylaxis programs, including the education of healthcare professionals on guideline adherence, standardization of antibiotic administration timing, and regular audit of prophylaxis practices (Rizzo et al. 2025). As demonstrated in European surgical settings, even in high‐resource environments, continuous attention to protocol compliance is essential for optimizing outcomes.

Second, the findings support prioritization of antimicrobial stewardship interventions targeting third‐generation cephalosporins and fluoroquinolones. Hospitals should implement prospective audit and feedback, formulary restrictions, or pre‐authorization requirements for these agents, particularly in surgical wards and intensive care units where resistance rates are highest. Educational interventions targeting prescribers should emphasize the time‐lagged relationship between antibiotic use and resistance, the limited oral options for resistant infections, and the importance of obtaining cultures before initiating therapy.

Third, infection prevention and control activities should be intensified in identified geographic hotspots (Cayo and Belize districts) and within high‐risk units of tertiary hospitals. Enhanced environmental cleaning, hand hygiene compliance monitoring, and contact precautions for patients with known resistant infections should be prioritized where resources permit. Given the association between specific procedures and resistant pathogens, preoperative decolonization protocols (e.g., for MRSA in amputation candidates) and targeted perioperative prophylaxis warrant evaluation.

Fourth, laboratory capacity strengthening should be prioritized to enable reliable detection of resistant organisms, including ESBL producers, MRSA, and emerging threats like CRE. Ensuring consistent application of CLSI standards, participation in EQA programs, and retention of isolates for future molecular characterization would enhance surveillance quality. The development of a national antibiogram, updated annually and stratified by facility and specimen type, would provide clinicians with accessible, locally relevant susceptibility data to guide empirical therapy.

Fifth, the findings underscore the need for continued national surveillance with expansion to include private sector facilities, outpatient settings, and additional pathogens. Real‐time or near‐real‐time surveillance platforms, integrated with the BHIS, could enable early detection of emerging resistance trends and rapid response to outbreaks.

### 4.5 Future Research Directions

This study identifies multiple avenues for future research. Prospective cohort studies with comprehensive patient‐level data collection (comorbidities, prior antibiotic exposure, healthcare contacts, surgical characteristics, outcomes) are needed to confirm procedure‐specific risk associations and quantify the clinical and economic burden of resistant infections. Interventional studies evaluating the impact of antibiotic stewardship programs, particularly those targeting third‐generation cephalosporins and fluoroquinolones, would provide causal evidence to guide policy. Molecular epidemiological studies, including whole‐genome sequencing of resistant isolates, would elucidate transmission dynamics, distinguish between clonal spread and horizontal gene transfer, and detect emerging resistance mechanisms. Research on community antibiotic use, including over‐the‐counter sales and prescribing practices in private clinics, would complete the picture of antibiotic selection pressure. Finally, implementation science research examining barriers and facilitators to guideline adherence, stewardship implementation, and infection control practice change in resource‐limited settings would inform strategies for translating evidence into practice.

## 5 Conclusions

This 7‐year national surveillance analysis provides evidence that AMR in Belize is prevalent and, for specific pathogen‐phenotype combinations, has increased over the study period. ESBL‐producing Enterobacteriaceae and MRSA constitute a substantial proportion of isolates from surgical site infections, with implications for empirical treatment selection. The identification of geographic and procedure‐specific risk factors offers opportunities for targeted interventions. The time‐lagged associations between antibiotic consumption and resistance support ongoing stewardship efforts. While sentinel detection of CRE and VRE warrants continued vigilance, these findings should be interpreted as preliminary signals rather than established prevalence. These findings collectively suggest that strengthening antimicrobial stewardship, infection control, and surveillance should be public health priorities. The data presented here provide a foundational evidence base upon which Belize can build a resilient defense to preserve the efficacy of essential medicines for future generations.

## Author Contributions

**Innocent E. Nwachukwu:** conceptualization, methodology, data collection, analysis, writing original draft preparation, review and editing, supervision, and project administration. **Cary W. Rivera:** conceptualization, methodology, data collection, analysis, writing original draft preparation, review and editing. **Vivian M. Reyes:** conceptualization, methodology, data collection, analysis, writing original draft preparation, review, and editing. **Diomar Salazar:** conceptualization, methodology, writing, review, editing, validation, supervision. **Lydia Harris‐Thurton:** methodology, writing, review, editing, validation, supervision.**Danladi C. Husaini:** conceptualization, methodology, data analysis, validation, writing original draft preparation, visualization, review & editing, and project administration.

## Funding

The authors have nothing to report.

## Ethics Statement

Ethics approval was received from the University of Belize IRB (Protocol #24‐I‐0009) and the Belize Ministry of Health and Wellness (GEN/147/01/24 (43) Vol. VI).

## Consent

The authors have nothing to report.

## Conflicts of Interest

The authors declare no conflicts of interest.

## Policy on Using ChatGPT and Similar AI Tools

During the preparation of this work the authors used Manus‐AI as part of the software to generate the Graphical Abstract. After using this tool, the authors reviewed and edited the content as needed and take full responsibility for the content of the published article.

## Data Availability

All the data generated and associated with this research has been provided in this article.
